# A high-resolution system for metabolic imaging of cancer

**DOI:** 10.1186/1753-6561-7-S2-O3

**Published:** 2013-04-04

**Authors:** Eduardo H Moriyama, Eric Leung, Naz Chaudary, Tuula Kalliomaki, Richard P Hill, Brian C Wilson, Michael Milosevic

**Affiliations:** 1 Division of Biophysics and Bioimaging, Princess Margaret Hospital, Ontario Cancer Institute, Toronto, ON, Canada; 2 Radiation Medicine Program, Princess Margaret Hospital, Ontario Cancer Institute, Toronto, ON, Canada; 3 The Campbell Family Institute for Cancer Research, Ontario Cancer Institute, Toronto, ON, Canada

## 

Alterations in metabolic signaling pathways allow tumors to achieve balance between oxygen supply and consumption via activation of several genes involved in adaptation to low oxygen conditions (hypoxia). Hypoxia stimulates glucose uptake, glycolysis and changes in mitochondrial respiration in tumors as a direct result of increased HIF-1 transcriptional activity. HIF-1α stabilization induces the expression of genes involved in cell survival, proliferation, angiogenesis and glucose transport (GLUTs) and the stimulation of genes involved in glycolytic energy production. Elevated anaerobic metabolism results in increased production of lactate as an alternate cellular energy source. Recent studies suggested that increased levels of lactate in tumors are associated with tumor progression, development of metastases, radioresistance and decrease of patient survival. Therefore, the ability to monitor products of altered metabolism in tumors would provide valuable information to improve cancer treatment.

We have developed a novel high-resolution imaging system to assess the distribution of metabolites in tumors such as lactate and ATP. Initial tests demonstrate the capacity of this technique in providing quantitative measurements of metabolites with high spatial resolution. This technology offers several important advantages over other techniques including ease of translation to the clinic, microscopic spatial localization and quantitation of metabolite concentration. Another advantage on the use of this technique is the possibility of spatial co-registration of metabolites with other tumors biomarkers. The main focus of this approach is the development of novel technologies for imaging tumor metabolism. This will lead to a better understanding of the role of tumor metabolism in treatment outcome, leading to more rationale therapeutic protocols.

## Financial support

The Terry Fox Foundation, Canada.

